# Virtual Reality–Based Executive Function Rehabilitation System for Children With Traumatic Brain Injury: Design and Usability Study

**DOI:** 10.2196/16947

**Published:** 2020-08-25

**Authors:** Jiabin Shen, Henry Xiang, John Luna, Alice Grishchenko, Jeremy Patterson, Robert V Strouse, Maxwell Roland, Jennifer P Lundine, Christine H Koterba, Kimberly Lever, Jonathan I Groner, Yungui Huang, En-Ju Deborah Lin

**Affiliations:** 1 Center for Biobehavioral Health Abigail Wexner Research Institute at Nationwide Children's Hospital Columbus, OH United States; 2 Center for Injury Research and Policy Abigail Wexner Research Institute at Nationwide Children's Hospital Columbus, OH United States; 3 Research Information Solutions and Innovation Abigail Wexner Research Institute at Nationwide Children's Hospital Columbus, OH United States; 4 Department of Speech & Hearing Science The Ohio State University Columbus, OH United States; 5 Inpatient Rehabilitation Program Nationwide Children's Hospital Columbus, OH United States; 6 Trauma Program Nationwide Children's Hospital Columbus, OH United States

**Keywords:** traumatic brain injury, virtual reality, neurological rehabilitation, executive function, cognitive rehabilitation

## Abstract

**Background:**

Traumatic brain injury (TBI) poses a significant threat to children’s health. Cognitive rehabilitation for pediatric TBI has the potential to improve the quality of life following the injury. Virtual reality (VR) can provide enriched cognitive training in a life-like but safe environment. However, existing VR applications for pediatric TBIs have primarily focused on physical rehabilitation.

**Objective:**

This study aims to design and develop an integrative hardware and software VR system to provide rehabilitation of executive functions (EF) for children with TBI, particularly in 3 core EF: inhibitory control, working memory, and cognitive flexibility.

**Methods:**

The VR training system was developed by an interdisciplinary team with expertise in best practices of VR design, developmental psychology, and pediatric TBI rehabilitation. Pilot usability testing of this novel system was conducted among 10 healthy children and 4 children with TBIs.

**Results:**

Our VR-based interactive cognitive training system was developed to provide assistive training on core EF following pediatric TBI. Pilot usability testing showed adequate user satisfaction ratings for both the hardware and software components of the VR system.

**Conclusions:**

This project designed and tested a novel VR-based system for executive function rehabilitation that is specifically adapted to children following TBI.

## Introduction

### Background

Opportunities to use virtual reality (VR) in health care are increasing as technology advances and accessibility to VR improves. VR provides a novel way to create an engaging virtual world to deliver authentically convincing simulations with measurable tasks in a controlled, low-risk environment. Current health care applications of VR include patient or provider education, pain or anxiety reduction, and therapeutic interventions [1-3].

Leveraging VR for the rehabilitation of cognitive and motor functions for children after a traumatic brain injury (TBI) is an active field of research and development [4-6]. TBI often disrupts the normal function of the brain of a child and is the leading cause of death and acquired disability in children, with an estimated 700,000 pediatric TBI cases annually in the United States [7,8]. Severe TBI in children often causes significant functional losses in memory, communication, and muscle control; long-term therapies are required to recover both cognitively and physically. Most traditional rehabilitation exercises involve repetitive, task-oriented training, which patients with TBI have reported to be boring, leading to poor adherence [9,10]. VR may provide a more engaging and cost-effective alternative or supplement to the traditional rehabilitation program [4-6].

Current therapeutic VR applications for pediatric TBI mostly focus on physical rehabilitation and include games that assist patients in regaining balance, increasing extremity strength, and practicing real-life tasks in low-risk environments [5,11-13]. Our systematic review of the use of VR in pediatric TBI rehabilitation found only 3 studies meeting the review criteria, and all of them focused on the positive effects of VR on physical rehabilitation [6]. However, it is promising to learn that VR’s efficacy in cognitive rehabilitation in adults with TBI has turned out positive. For instance, Grealy et al [14] found that exercise-based VR rehabilitation increased adult patient performance on visual and verbal learning tasks as compared with patients in traditional rehabilitation programs. Jacoby et al [15] reported that adults with TBI in VR-based task-specific therapy performed better on executive function tests than patients in therapy without VR. Finally, Caglio et al [16] found that adult patients who used VR for navigational tasks increased their memory capacity.

### Objectives

The goal of this study was to design a VR system specifically for executive function (EF) rehabilitation among children with TBI. Few VR systems have been reported in this domain, with even fewer designed specifically to meet the physical, psychological, and medical needs of this vulnerable population. Potential complications of pediatric TBI may dictate the design of an appropriate VR-based cognitive rehabilitation solution for this patient population. For example, most of the current VR systems rely on a head-mounted display (HMD) and require the patient to move his or her head to navigate in the VR environment, which can be unsafe for patients with severe head injuries due to possible skull fractures or scalp sutures. During the process of addressing these safety concerns and developing a VR-based interactive cognitive training system (referred as *the VR system* in the remainder of the paper) to facilitate EF rehabilitation in children with TBI, we identified several useful approaches that may inform the future development of VR applications with similar challenges. This paper presents the specific design elements of the VR system to highlight these practical considerations.

## Methods

### Design and Development of the Virtual Reality System

Developing a VR solution typically involves many steps and components, including hardware selection and customization, the development and testing environment, defining outcome metrics, end-user interaction design, administrator control design, and data collections and analytics. This section provides a detailed review of the major components of the VR solution.

#### Hardware Options of the Virtual Reality System

A variety of devices and components can deliver the VR experience and build VR software. In most cases, highly desired features (eg, lightweight and positional tracking) should be considered in concert with the proposed utility and the available resources. Currently, the main categories to consider are smartphone VR headsets, tethered PC-based VR headsets (eg, HTC Vive), or standalone VR headsets (eg, Oculus Quest; Table 1). Smartphone VR headsets deliver the VR experience through a smartphone fitted on a headset that can be as simple as the original Google Cardboard. Although these types of VR applications are more cost-effective and easier to disseminate, resolution, frame rate, and insufficient input mechanisms supporting user interaction limit the delivered VR experience as compared with the higher end PC-based or standalone VR headsets. The tethered VR systems include a headset that is physically or wirelessly connected to a computer. A high-quality headset connected to a powerful gaming PC tends to provide the most immersive VR experience because of the high tracking accuracy and superior graphics quality. Console VR, which is currently limited to Playstation VR, offers features similar to PC-based systems. Unlike PC-based VR, Playstation VR is powered by a Sony Playstation 4 video game console. Positional tracking is performed by a single Playstation camera, and Playstation Move controllers are used for input. The standalone VR headsets (also referred to as all-in-one VR headsets) have built-in processors, sensors, batteries, storage memory, and displays. These systems are wireless and easy to use and typically offer a VR experience of a quality between that provided by the smartphone VR and PC-based VR. Consequently, current technology leaders in VR are focusing more on the design of headsets in this category, and wireless yet powerful VR headsets are likely to dominate soon. Recent offerings such as the Oculus Quest and Vive Focus Plus already adopt the wireless inside-out tracking to provide 6 degrees of freedom (DoF) and have the potential for unlimited movement.

**Table 1:** Comparison of commercially available virtual reality systems.

Type of headset	Advantages	Disadvantages	Examples
Smartphone VR^a^	Low cost (ie, viewer using an existing smartphone) Easier set up Scalability	Low-fidelity graphics Increased work to achieve optimization Three DoF input Requires compatible smartphone Increased motion sickness risk	Samsung GearVR Google Daydream^b^ Google Cardboard
PC-based VR	Immersive presence Precise 6 DoF^c^ tracking (ie, orientation and position) Room scale Allows external monitoring High fidelity	Heavy Tethered to PC Advance setup (outside-in only) Loss of hand tracking (inside-out only)	Oculus Rift S HTC Vive Suite Valve Index Windows Mixed Reality
Console VR	Comfortable headset Less expensive than gaming PC	Requires Sony development license and DevNet access Loses tracking easily Tethered to console	Playstation VR
Standalone VR	Portability Easier set up Cheaper to deploy compared with the console or PC-based VR	Low-fidelity graphics Increased work to achieve optimization No or limited movement on 3 DoF hardware	Oculus Quest Vive Focus Plus Oculus Go (3 DoF only) Lenovo Mirage VR S3 (3 DoF only)

^a^VR: virtual reality.

^b^Google recently announced that they will not sell the Daydream viewer and will not include Daydream compatibility in phones going forward. Existing Daydream devices will still have access to the Daydream platform at the time of writing.

^c^DoF: degrees of freedom.

#### Hardware of the Virtual Reality System

**Figure 1:**
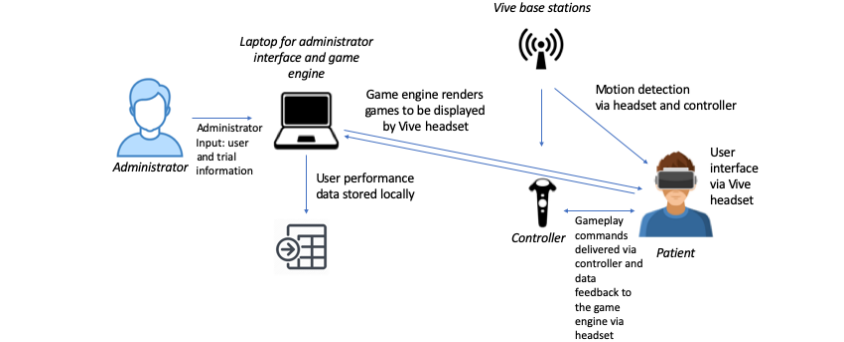
System component diagram showing data flow and user interaction.

For the purpose of this study, we chose to use a PC-based VR program. The system consists of an HTC Vive VR headset and Vive controller (both by HTC Vive Tech Corporation), an Alienware laptop (Dell Inc), a customized portable station, and 2 infrared projectors with tripods. Figure 1 depicts the interactions of the system components. The HTC Vive system and an Alienware laptop were used as they were tailored for gaming with superior real-time graphics rendering. At the time of its initial development in 2016, the Vive offered the best field of vision and resolution with the most reliable positional tracking. For positional tracking, the Vive base stations emit alternating infrared pulses and laser sweeps at 60 times per second. The photosensors on the headset and controllers use the timing difference between the infrared and lasers to determine the position and orientation with submillimeter precision. A top-of-the-line gaming laptop, such as hardware from Alienware, reduces the likelihood of VR simulation sickness by rendering more complex real-time graphics at a higher frame rate [17], while also allowing for increased portability, as compared with a desktop PC.

A Vive controller input device was used to provide the user with a physical connection to the virtual world. The game displayed the Vive controller as a virtual hand with which the user presses virtual buttons. By seeing their hand movements represented directly in the virtual world, the user had a stronger sense of presence (ie, the sense of being in the virtual environment), thus leading to a more immersive experience [18-20]. However, one important lesson learned was that the large size of the Vive controller relative to the small hands of the younger users led to their use of both hands to handle the controller. This dual hand usage could have led to a dissociation of the one-to-one hand representation for some users.

**Figure 2:**
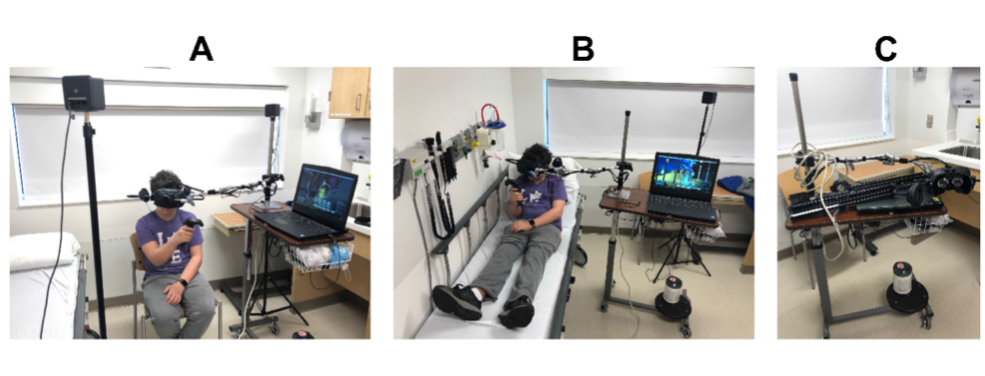
Virtual reality–based interactive cognitive wheeled workstation. Operated with a child sitting in a chair (left). The child operating the program while reclining in bed (middle). Equipment is sanitized after each use and stored as a compact and portable workstation (right).

One unique consideration in designing a VR system for pediatric TBI rehabilitation is minimizing the headset weight upon the child’s head when donning the HMD. As the VR headset can weigh as much as 0.83 kg (0.56 kg in the case of the HTC Vive), some secondary effects of a TBI (eg, skull fracture and scalp sutures) might preclude direct head mounting. To circumvent this issue, we custom-mounted the VR headset to an adjustable mechanical arm attached to a cart (Figure 2). Also, a headphone secured on the sides of the headset reduced direct contact and weight on the head. The headset is hence positionally and rotationally fixed and does not weigh down on the head. The mechanical support system was designed to accommodate users in both sitting and reclining positions. This allowed users to experience VR in a chair or in their hospital bed. In this setup, 2 infrared projectors placed on either side of the user served to detect the position of the controller relative to the VR space. A detailed description of the custom-mount setup is provided in Multimedia Appendix 1.

#### Software of the Virtual Reality System

##### Development Environment

The increasing accessibility of VR affords many software choices for developing VR, such as Unity3D, Unreal Engine, AppGameKit VR, CryEngine, Amazon Lumberyard, and ApertusVR. The choice of software is the developer’s preference as they are comparable. For the VR system described in the study, the game contents were developed using the Unity game engine (Unity Technologies). Maya 3D software (Autodesk) was used for 3D modeling and animation, and Photoshop (Adobe) was used to create 2-dimensional assets.

##### Interface for the Researcher or Therapist

As cognitive rehabilitation is a long-term repetitive process, designing an interface that allows the therapist to enter patient and session information to track progress is an important consideration. Enabling specific controls over the game, such as stop and restart, and options to choose different modules is also advantageous. Likewise, the ability to observe patients as they play the game may give the therapist insights into their progress and deliver timely feedback or support.

**Figure 3:**
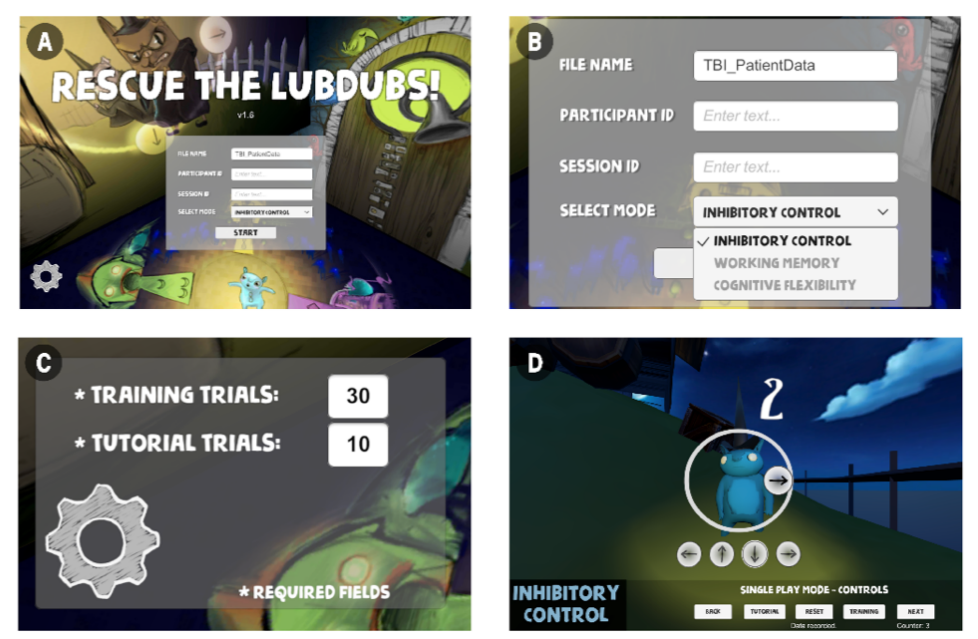
User-friendly interface for therapist. (A) Enter user ID and session ID. (B) Choose training module to run. (C) Customize training by changing number of trials. (D) Control, track, and instruct using laptop trackpad or use a second controller.

One of the advantages of a PC-tethered system (eg, Vive) is that it easily accommodates a separate interface for the therapist without needing to connect separate devices over a server. Through the main interface for the VR system (Figure 3), the therapist can enter user information and session IDs, select the training module, customize training by setting the number of trials the user will play, and monitor VR training progress. The therapist can control the progress of the game on the interface with 5 buttons—*Back* (go back to the home screen), *Tutorial* (a short version of the trial, data not collected), *Reset* (interrupts the trial and resets the trial to its initialized state), *Training* (full-version trial with data collection), and *Next* (go to the next game). The therapist can directly instruct the user in the tutorial module by drawing in the virtual space using the trackpad on the laptop or a second controller.

#### Virtual Reality Game Design for Executive Function Training

### Overview

Game design is a crucial element in creating an effective VR cognitive rehabilitation system. Cognitive exercises with adjustable levels of difficulty are required to meet the varied needs of TBI users. More in-depth reviews of various game designs for cognitive training are available for the interested reader [21-23]. In this section, we present the development of the VR system to highlight some design considerations.

Development of our VR system was focused on executive function rehabilitation because pediatric TBIs, especially moderate to severe cases, often result in executive dysfunction due to the vulnerability of the frontal lobes. One theoretical rationale underlying the mechanism for repeated VR-based tasks to have a potential training effect on the EF of children with TBI beyond the trained tasks is that EF are mostly regarded as domain-general skills, as opposed to domain-specific skills, in both a healthy population [24,25] and patients with TBI [26,27]. Following this rationale, the VR system includes 3 VR games for training 3 core EF: game 1 for *inhibitory control* (the ability to override a strong internal predisposition or external lure and do what is more appropriate or needed), game 2 for *working memory* (the ability to hold and process information in mind as needed), and game 3 for *cognitive flexibility* (the ability to adjust to changing environmental demands and think from different perspectives).

### Story Narrative

**Figure 4:**
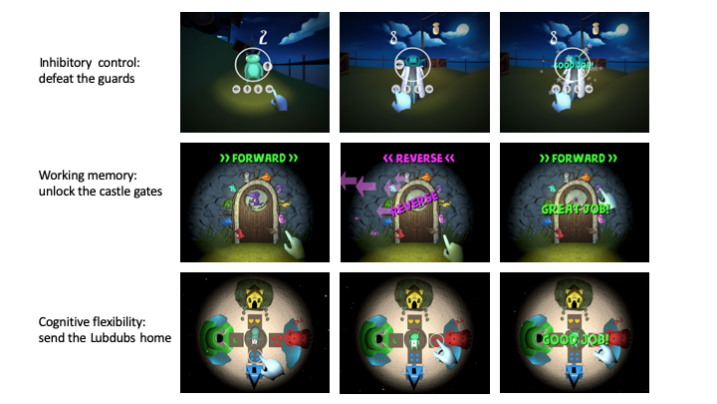
Virtual reality–based interactive cognitive training modules. (Top panel) inhibitory control, (middle panel) working memory, and (bottom panel) cognitive flexibility.

A useful and increasingly popular approach in digital game design is the use of a story narrative [28-30]. A story narrative creates a more meaningful and immersive experience for the user. A narrative background helps the user to feel more involved and thus enhances engagement. In addition, background narrative is a mechanism that conveys the perceived conceptual depth of the virtual environment to the user before active engagement begins, thus reinforcing the user’s sense of realism. The story narrative, presented at the start of the game, is best delivered through voice narration rather than text, to improve access for a younger target audience. As illustrated in Figure 4, the story narrative for the VR system is a mission to *Rescue the Lubdubs* and presented by the research staff as follows:

Lubdubs are magical creatures that live in a different world. They have been captured, and your job is to return them safely to their homes. You will play three mini-games. Our goal is to get through all the guards of the castle by completing each of the three games and rescuing the lubdubs within the castle.

### Game 1: Inhibitory Control

This game design was based on a classic psychological task for inhibitory control, the Spatial Stroop Task [31], which is used to train and assess inhibitory control. The Spatial Stroop Task examines the ability of the user to respond correctly to the interference between the stimulus location with the location information in the stimuli. In this game, the user is *battling* different characters. An arrow appears randomly on 1 of the 4 positions in relation to the character the user is *battling* (ie, above, below, to the left, or to the right). The direction of the arrow may or may not be the same as its *position* relative to the character. The user is required to tap on the arrow below that matches the arrow that appears on the circle in the middle of the screen. Inhibitory control is the ability to override a strong internal predisposition or external cues and do what is more appropriate. Thus, in this game, the user needs to ignore the *positional* cue (eg, right of the character) and respond to the actual direction of the arrow (eg, up arrow, as depicted in Figure 4 top panel). To maintain interest and engagement, the user will move to a different character after a few rounds, as if progressing through a series of battles. The data collected for this module include time taken to respond, arrow position and direction, user response (arrow direction), and correctness of the selection.

### Game 2: Working Memory

This game is a sequence recall game for training working memory that is adapted from the Visual Working Memory Task [32]. This game consists of a locked door with different characters around the door (Figure 4 middle panel). To *unlock* the door, the user needs to remember the order of characters displayed on the center of the door. The game starts with a sequence of 2 characters and adjusts difficulty level based on the responses of the user. Every time the user gets 2 consecutive sequences correct, the sequences increase in length by 1 character.

Conversely, if 2 consecutive sequences are incorrect, the sequences reduce in length by 1. Also, the trial will ask the user to recall the displayed sequence either in forward or reverse order. This reverse task is an essential component of working memory training. By definition, working memory is a system for temporary information storage for the execution of more complex cognitive tasks such as reasoning and information manipulation. The reverse task is designed to require an additional level of information processing.

To sustain interest, the user progresses through 5 different doors, with different characters, performing one-fifth of the total trials at each door. The sequence length carries over from the previous trial (ie, does not reset at each door) to ensure the trial will be sufficiently challenging to achieve the training purpose. The data collected for the module include time to submit the sequence, length of the presented sequence, length of the user sequence, and accuracy of the sequence.

### Game 3: Cognitive Flexibility

This game on cognitive flexibility is adapted from the Wisconsin Card Sorting Task [33,34]. In this task-switching paradigm, the user has to figure out which rule underlies a task (eg, match by color, shape, or number) and determine when the rule changes. In the VR system, to send the Lubdubs back to their homes, the user has to figure out what sorting method is being used to match the symbol on the Lubdub’s stomach to one of the four symbols in front of the houses (as shown in Figure 4 bottom panel). Every choice will result in a correct or incorrect feedback from the program to the user, and the user will need to determine the current rule based on the response. The rule is set to change every 7 trials, although this is undisclosed to the user. The data collected for this game include the time taken to respond, presented icon, current matching rule, user choice, and the accuracy of the answer.

#### Usability Testing of the Virtual Reality System

We conducted feasibility exercises of the VR system (the same version as used for patient population) on a convenience sample of healthy volunteers before testing on users with TBI. As the data collected were intended to be used as feedback data for the developers to improve the design of the VR systems rather than for human research purposes, the institutional review board (IRB) at Nationwide Children’s Hospital agreed that IRB review was not required. This feasibility exercise among healthy volunteers allowed us to better understand and potentially reduce the likelihood of negative effects on the pediatric users with TBI who may have been more sensitive to particular sensory effects (eg, simulation sickness and discomfort due to sound and light effects). In total, 10 healthy children aged between 7 and 17 years (mean age 14.30 years, SD 3.56 years) recruited from local communities in a midwestern urban city participated in this feasibility exercise. After playing for 10 min (which was sufficient time to try out all 3 games, although they were not required to complete all trials of each game as per the goal of this feasibility exercise), the users filled out the Simulation Sickness Questionnaire (SSQ) [35]; the Borg Perceived Physical Exertion Scale [36]; and a brief custom-made VR experience survey with questions on pleasure, motivation, and realism (Multimedia Appendix 2). For each of the questions, the users were asked to rate their responses from 0 (not at all) to 100 (very much). We also examined how patients with TBI completed the tasks, the time taken to complete the tasks, and the accuracy of responses. The protocol for collecting data from patients with TBI was reviewed and approved by the IRB, and informed consent or assent was obtained from the patients and their legal guardians before participating in study activities. A total of 4 pediatric patients with moderate to severe TBI as determined by the Glasgow Coma Scale (mean 5.00, SD 3.05) were recruited from an inpatient rehabilitation unit in a level I trauma center in a midwestern urban city. The patients were aged between 7 and 17 years (mean 11.75 years, SD 2.60 years), and all of them completed up to 50 trials for each of the 3 VR tasks and the same set of usability surveys as the healthy users.

Data collected by the VR system (Table 2) are stored locally on a password-protected laptop in a csv file without Protected Health Information connected to any of the data. These raw data were used to derive the response time to complete each trial and the percentage of correct responses.

**Table 2:** Metrics data automatically collected by virtual reality–based interactive cognitive training.

Task and metrics^a^			Possible values
**Inhibitory control**			
	Location of arrow	(up or down or left or right)	
	Direction of arrow	(up or down or left or right)	
	Condition	(consistent or inconsistent)	
	Response	(up or down or left or right)	
	Response time	(minutes:seconds:milliseconds)	
	Correct?	(yes or no)	
**Working memory**			
	Order	(forward or backward)	
	Number of items	(provide range)	
	Level	(provide range)	
	Response time	(minutes:seconds:milliseconds)	
	Correct?	(yes or no)	
**Cognitive flexibility**			
	Rule applied	(amount or shape)	
	Amount or color or shape	(amount or color or shape)	
	Response	(amount or color or shape)	
	Response time	(minutes:seconds:milliseconds)	
	Correct?	(yes or no)	

^a^For each training session, the study ID and the session number are entered by the researcher and are stored along with the metrics collected for the 3 training modules.

## Results

### Testing in Healthy Children

As shown in Table 3, healthy children reported a high level of fun and enjoyability. In the context of therapy, participants reported that they would highly desire that the VR system be available at hospitals and would be highly motivated to attend postdischarge therapies if they were ever to have a TBI. The participants reported low levels of simulation sickness on the SSQ and felt very light exertion due to playing the VR games.

**Table 3:** Usability findings in healthy children and children with traumatic brain injury.

Measure	Healthy children (n=10), mean (SD)	Children with TBI^a^ (n=4), mean (SD)
How realistic did you feel about the virtual reality environment? (0-100)	58.8 (35.6)	37.5 (47.9)
How much fun did you feel about the virtual reality games you just played? (0-100)	72.0 (23.9)	73.5 (31.5)
How did you like the virtual reality games you just played? (0-100)	72.0 (25.8)	67.5 (39.5)
Do you want to play them again in future? (0-100)	72.5 (26.8)	56.3 (43.9)
Do you want to have such virtual reality games in your future therapies while you are in hospital? (0-100)	85.0 (22.1)	50.3 (52.5)
Would you be more motivated to attend your therapy sessions after discharge if we include such games? (0-100)	87.0 (19.9)	41.5 (49.9)
Simulator sickness (0-48)	1.60 (1.07)	2.50 (2.08)
Physical exertion (6-20)	8.25 (1.63)	9.9 (2.3)

^a^TBI: traumatic brain injury.

As seen in Table 4, the healthy children were generally able to complete the trials in a reasonable amount of time (averaging less than 4 seconds per trial). The percentage of correct responses indicated that healthy children were able to complete inhibitory controls with high accuracy, whereas the other 2 tasks, working memory and cognitive flexibility, proved to be more challenging.

**Table 4:** Virtual reality–based interactive cognitive training performance of healthy children and pediatric patients with traumatic brain injury.

Task	Number of trials completed, range			Average time per trial, seconds, mean (SD)			Proportion of correct responses^a^ (%), mean (SD)	
	Healthy children^b^ (n=8)	Children with TBI^c^ (n=4)	Healthy children^b^ (n=8)		Children with TBI (n=4)	Healthy children^b^ (n=8)		Children with TBI (n=4)
Inhibitory control	11-50	50^d^	3.2 (5.5)		4.9 5.6)	98 (3.2)		95 (7.6)
Working memory	10-34	30-50	3.9 (1.1)		11.8 (9.2)^e^	74 (5.5)		50 (17.1)
Cognitive flexibility	32-50	50^d^	2.7 (1.6)		6.2 (4.7)	71 (11.9)		59 (9.2)

^a^Percentage of correct responses is calculated as the number of trials with correct responses over the total number of trials completed for the task by the subject.

^b^Data were not captured properly for 2 out of the 10 healthy volunteers, and thus, they were omitted.

^c^TBI: traumatic brain injury.

^d^Everyone completed 50 trials.

^e^Two users completed only 30 trials of the working memory task due to technical issues of the system, not due to their inability to complete.

### Testing in Children With Traumatic Brain Injury

Our preliminary testing in 4 children with TBI indicates that children with TBI can complete all 3 games, although requiring longer time for each trial (Table 4). On the basis of the trial response time captured in the app, the cumulative completion time for 50 trials ranged from 63.0 to 670.1 seconds for the inhibitory control task, 347.9 to 1283.5 seconds for the working memory task, and 92.2 to 537.7 seconds for the cognitive flexibility task. However, the loading and feedback time between trials was not captured, so the actual time taken to participate in the training was longer. There were significant subject variations in task performance, highlighting substantial variability in the cognitive ability of the patients (also reflecting the small sample size of this pilot testing), which is to be expected due to the wide range of effects of TBI on different individuals. The working memory task appears to be the most challenging among the 3 tasks, with low accuracy scores and long response times.

In the pilot study, the number of trials for each task was set at 50 by the researchers. This number was based on the estimated time taken to complete each trial for children within reasonable tolerance levels. The intention was to set the number of trials at a level achievable within a reasonable amount of time (15 min) to prevent overfatigue and disengagement. Concurrently, it should also be long enough to achieve the *training* purpose.

With the variation in the cognitive ability in children with TBI, it would be useful to include features for the therapists to adjust the number of trials and/or difficulty of training for an individual patient so that training is sufficiently challenging yet achievable (Figure 3) [10]. The optimal training remains to be evaluated in clinical studies and is outside the scope of this paper.

Children with TBI also completed the same questionnaires on usability and the SSQ. As with healthy subjects, they reported low levels of simulation sickness on the SSQ and felt very light exertion due to playing the VR games (Table 3). Interestingly, although children with TBI also reported a high level of fun engagement with the VR games, their attitude toward future use in therapy was highly variable. This may be, in part, due to the rehabilitative nature of the games being more challenging for these children, as indicated by longer trial completion time and lower percentage response accuracy (Table 4), in addition to the small sample size. Notably, some children were anecdotally comparing the games with commercial video games and not with the current rehabilitative programs. Future studies should assess children’s preference for VR rehabilitation against standard rehabilitation programs.

For both healthy and pediatric TBI groups, along with the structured quantitative assessment, this study used unstructured verbal feedback by the children and observation during the testing to inform further adjustment to the system. For example, spacing for selection choices in the *working memory* game was observed to be too narrow for some children and was adjusted to reduce frustration in aiming for their intended selection. It was observed that unintentional selection was made when the controller hovers over the selection choices. To address this, the control mechanism changed to mimic the movement of the hand actually needed to represent pressing a physical button. This was later replaced again to a laser pointer style *click* to reduce the complexity. It was also observed that children with smaller hands tend to hold the controller with both hands and thus have a different way of interacting with the controller. This made it hard for these children to reach the trigger button on the Vive controller, so we duplicated all trigger button functions to the trackpad button.

## Discussion

### Design Considerations for Virtual Reality−Based Cognitive Rehabilitation in Pediatric Patients With TBI

The development of the VR system offered a unique cross-disciplinary perspective, incorporating expertise from professionals working in developmental psychology, digital health, and pediatric rehabilitation. A user-centered design philosophy was implemented to create custom-developed hardware and software systems that indicated a high degree of usability for pediatric patients following a TBI. Textbox 1 summarizes a series of practical considerations specific to designing VR systems for pediatric TBI cognitive rehabilitation that we encountered and solved in developing this system.

**Design considerations for virtual reality applications used for traumatic brain injury rehabilitation.**

**Headset burden on the injured head**

- Mounted virtual reality (VR) headset on mechanical arm frees pediatric patients with traumatic brain injury (TBI) from physical burden

**VR simulation sickness**

- Mounted headset allows the virtual world to remain relatively static to minimize simulation sickness for pediatric patients with TBI as compared with the traditional 360° VR environment
- Dimming the peripheral view and “lighting up” the center of the gaming screen reduces the vision field
- A high-quality tracking system and high frame rate of the graphics help reduce motion sickness

**Engagement or replayability**

- Games designed with varying levels of difficulty help engage patients with different baseline skills and gaming experience
- Built-in procedurally generated design elements increase engagement and replayability by introducing near-infinite variety to each gameplay session. Example: various components of a character (eg, head, face, body, and color) can be randomly assembled to generate many different characters quickly

**User experience**

- Spacing between selection options can affect the user experience. This ensures users can easily select an answer

**Rehabilitation factors**

- Evidence-based rehabilitation theories in cognitive and developmental psychology and other fields guided the game designs
- Data collection and analysis were used, which are critical to track progress in long-term rehabilitation programs
- Adjustable levels of training matched with individual needs and progress to allow for improving functions in rehabilitation settings

**Privacy and data security**

- Implemented best practices in personal health data storage and security based on the data collected to ensure the highest level of privacy and security

### Limitations and Future Directions

This study has several limitations that we hope to overcome in future studies. First, this study used a convenience sample of healthy children and pediatric patients with TBI, which may not be representative of the target population in age and gender distributions. Second, this study was intended to collect usability data rather than outcome evaluation; therefore, we did not test out the feature of changing number of trials as a way to adjust the level of training difficulty. Third, for children with TBI, the initial intention was to have the subject complete the trials in a single session. However, in practice, due to the preferences of patients and time constraints from their other appointments, it was more feasible to complete all trials in multiple sessions. However, this information was not collected or analyzed in this preliminary study. Future studies of formal efficacy evaluation should consider adding this information in their analysis. Finally, future research should consider design features that are specific for patients in different age groups and with gaming experiences (eg, developing different story narratives) or that have varying number of training components for different subgroups of the pediatric population to increase both the level of engagement and potentially the training efficacy of the program. A pilot randomized clinical trial is currently underway to evaluate the preliminary efficacy of the VR system.

### Conclusions

VR offers an exciting approach for pediatric TBI cognitive rehabilitation. This tutorial describes the challenges faced, solutions to these problems, and lessons learned through the development of the VR system. With rapid advances in VR technology and accessibility, we believe there is significant potential to expand the current program for future telemedicine or in-home applications. However, more studies are needed to refine the design of these technologies and evaluate their feasibility and efficacy.

## Appendix

Multimedia Appendix 1 Detailed hardware configurations on the VR mount setup.

Multimedia Appendix 2 Brief VR Experience Survey.

## Abbreviations

EF: executive functions
HMD: head-mounted display
IRB: institutional review board
SSQ: simulation sickness questionnaire
TBI: traumatic brain injury
VR: virtual reality
