# Supplementary material for: Virtual Reality–Based Executive Function Rehabilitation System for Children With Traumatic Brain Injury: Design and Usability Study
Source: JMIR Serious Games. 2020 Aug 25;8(3):e16947. doi: 10.2196/16947 (PMC7479584; doi:10.2196/16947)
Supplement: Multimedia Appendix 1 [file games_v8i3e16947_app1.docx]

**SUPPLEMENTARY MATERIALS**

**Custom Mount Setup**

The custom mount setup was made from a combination of COTS (commercial-off-the-shelf) products and custom fabricated parts. Commercial-off-the-shelf products include several products related to photography lighting and rigging.

Specifically, A 1” diameter, 24” long, 12 gauge stainless steel tube was welded to a ¼” thick 6”x6” stainless steel plate. 4 1 ½” long ¼-20 stainless steel all threads were welded to the top of this plate approximately ¾” on center in from both sides of each corner. Another ¼” thick 6”x6” stainless steel plate was fabricated with corresponding 5/16”D holes approximately ¾” in from each corner. Clearance holes were drilled through the carts wooden horizontal surface for the 1” stainless steel tube and ¼” all threads. The two places were then placed on either side of the horizontal surface and secured with ¼-20 stainless steel acorn nuts. A small mounting bracket was designed and 3D printed to serve as an interface between the camera rigging attachment arm and the front of the VR headset. The VR headset was reverse engineered in CAD and a mounting location was identified. This mounting location served as the inside surface of the small mounting bracket while standard camera rigging geometry was created to serve as the outside of the bracket. The small mounting bracket was adhered to the front of the VR headset using a two-part epoxy designed to bond plastics together.

List of hardware components that were purchased as COTS:

1. Amazon Basics Aluminum Light Photography Tripod Stand with Case - Pack of 2, 2.8 - 6.7 Feet, Black (<https://www.amazon.com/AmazonBasics-Aluminum-7-Foot-Light-Stand/dp/B074VMTP68>)

2. Manfrotto 196B-2 143BKT 2-Section Single Articulated Arm with Camera Bracket (Black) (<https://www.amazon.com/Manfrotto-196B-2-2-Section-Articulated-Bracket/dp/B000OPWSYM>)

3. EMART Photo Video Studio Heavy Duty Metal Clamp Holder with 5/8" Light Stand Attachment and Umbrella Reflector Holder (<https://www.amazon.com/EMART-Studio-Attachment-Umbrella-Reflector/dp/B07QYKH7K3>)
