# Supplementary material for: Virtual Reality–Based Executive Function Rehabilitation System for Children With Traumatic Brain Injury: Design and Usability Study
Source: JMIR Serious Games. 2020 Aug 25;8(3):e16947. doi: 10.2196/16947 (PMC7479584; doi:10.2196/16947)
Supplement: Multimedia Appendix 2 [file games_v8i3e16947_app2.pdf]

SUPPLEMENTARY MATERIALS

Brief VR Experience Survey

<u>Realism</u>

How realistic did you feel about the virtual reality environment?

1————————————————1

Not at all 0 VAS (mm) 100 **Very much**

<u>Pleasure</u>

How much fun did you feel about the virtual reality games you just played?

1————————————————1

Not at all 0 VAS (mm) 100 **Very much**

<u>Motivation</u>

How did you like the virtual reality games you just played?

1————————————————1

Not at all 0 VAS (mm) 100 **Very much**

Do you want to play them again in future?

1————————————————1

Not at all 0 VAS (mm) 100 **Very much**

Do you want to have such virtual reality games in your future therapies while you are in hospital?

1————————————————1

Not at all 0 VAS (mm) 100 **Very much**

Would you be more motivated to attend your therapy sessions after discharge if we include such games?

1————————————————1

Not at all 0 VAS (mm) 100 **Very much**
